# Controlling the Functional Properties of K_0.5_Bi_0.5_TiO_3_ Ceramics Using E-Poling

**DOI:** 10.3390/ma19010034

**Published:** 2025-12-21

**Authors:** Jan Suchanicz, Marcin Wąs, Bartosz Handke, Piotr Jeleń, Zofia Kucia, Antoni Kania, Dorota Sitko, Kamila Kluczewska-Chmielarz, Krzysztof Konieczny, Jakub Gajda, Aleksander Zawada, Marcin Lapinski, Barbara Swatowska, Dagmara Brzezińska, Jakub Fitas, Tomasz Hebda, Grzegorz Stachowski

**Affiliations:** 1 Department of Mechanical Engineering and Agrophysics, University of Agriculture in Krakow, Balicka 120, 31-120 Krakow, Poland; jakub.fitas@urk.edu.pl (J.F.); tomasz.hebda@urk.edu.pl (T.H.); 2 Department of Bioprocess Engineering, Power Engineering and Automation, University of Agriculture in Krakow, Balicka 120, 31-120 Krakow, Poland; 3 Faculty of Materials Science and Ceramics, AGH-University of Science and Technology, al. Mickiewicza 30, 30-059 Kraków, Poland; bhandke@agh.edu.pl (B.H.); pjelen@agh.edu.pl (P.J.); zofiakucia@agh.edu.pl (Z.K.); 4 A. Chelkowski Institute of Physics, University of Silesia in Katowice, 75 Pułku Piechoty, 41-500 Chorzów, Poland; antoni.kania@us.edu.pl; 5 Faculty of Exact & Natural Sciences, University of the National Education Commission, Podchorazych 2, 30-084 Krakow, Poland; dorota.sitko@uken.krakow.pl; 6 Institute of Technical Sciences, University of the National Education Commission, Podchorazych 2, 30-084 Krakow, Poland; kamila.kluczewska-chmielarz@uken.krakow.pl (K.K.-C.); krzysztof.konieczny@uken.krakow.pl (K.K.); d751420@doktorant.uken.krakow.pl (J.G.); aleksander.zawada@uken.krakow.pl (A.Z.); 7 Institute of Nanotechnology and Materials Engineering, Advanced Materials Center, Gdansk University of Technology, 80-233 Gdańsk, Poland; marcin.lapinski@pg.edu.pl; 8 Institute of Electronics, AGH-University of Science and Technology, al. Mickiewicza 30, 30-059 Krakow, Poland; swatow@agh.edu.pl; 9 Faculty of Science and Technology, Institute of Materials Engineering, University of Silesia in Katowice, 75 Pułku Piechoty 1a, 41-500 Chorzow, Poland; dagmara.brzezinska@us.edu.pl; 10 Astronomical Observatory, Jagiellonian University, Orla 171, 30-244 Krakow, Poland; grzegorz.stachowski@uj.edu.pl

**Keywords:** lead-free, ceramics, E-poling, KBT

## Abstract

Lead-free K_0.5_Bi_0.5_TiO_3_ (KBT) ceramics were prepared using a finely tuned convectional solid-state reaction method. Their phase transitions in unpoled and poled states were examined. The temperature-dependent evolution of the reflections sensitive to structural changes and their 2Θ-positions indicated two temperature-driven phase transitions: tetragonal–tetragonal at about 200 °C, and tetragonal–cubic at around 400 °C. These structural transformations are further corroborated by studies examining Raman spectroscopy, dielectric properties, and mechanical properties. It was demonstrated that a prior E-field poling process significantly influences the polar state, causing an increase in the local degree of order, as well as the transformation of the cubic phase into the tetragonal one. This stabilizes and widens the temperature range of the ferroelectric phase. It was found that phase transformations in KBT are accompanied by a softening of the mechanical behavior similarly to improper ferroelastic transformations. The results demonstrate that KBT possesses favorable structural, dielectric, and mechanical characteristics, making it a potential candidate for electronic applications. The present study provides a clear understanding of the multi-scale structural behavior in multi-phase KBT, bridging micro-heterogeneity behaviors and macro-properties, and demonstrates an effective method of tuning the properties of KBTs by E-poling with a low electric field.

## 1. Introduction

Recently, significant attention has been directed towards research on lead-free ferroelectric materials such as BaTiO_3_, (K,Na)NO_3_, Na_0.5_Bi_0.5_TiO_3_, and K_0.5_Bi_0.5_TiO_3_-based systems due to concerns about the harmful environmental effects of lead [1,2,3]. Lead has a toxic nature and it persists in the environment for a long time, which is why its usage in electronic products is restricted by the European Union. In this context, the mentioned lead-free materials are suitable alternatives. K_0.5_Bi_0.5_TiO_3_ (KBT) is the most promising lead-free material due to its a relatively high Curie temperature and large strain [4,5,6]. KBT-based multilayered actuators can efficiently work under unipolar driving up to 100 kV/cm, even when using Ag-Pd electrodes as inner electrodes [6]. Recently, high energy storage performances and high breakdown strength were obtained via creating solid solutions of KBT with other perovskites or modifying their cation site [7,8]. Similar results were obtained via low electric field application [9] and multi-scale synergistic design [10]. These results showed that KBT has clear advantages as the starting point for obtaining materials with excellent energy storage performance and actuation.

KBT is a complex A’_0.5_B’_0.5_TiO_3_ perovskite exhibiting the random occupation of the A-site by the K^+^ and Bi^3+^ ions [11,12]. This structural disorder leads to the diffuse nature of phase transitions and can result in excellent application properties of this type of material. KBT exhibits either tetragonal symmetry [13] or a coexistence of tetragonal and cubic phases [14] at room temperature. Earlier studies identified two phase transitions from tetragonal to pseudocubic at about 200 °C [15] (or about 270 °C according to Ivanova et al. [16]) and then to cubic phase. According to Suchanicz et al. [14], there are two phase transitions: (1) between tetragonal phases within the temperature interval of 200–225 °C and (2) between tetragonal and cubic phases near 400 °C. Research on KBT remains limited due to the technological difficulties in synthesizing this compound, primarily because of the volatility of the potassium and bismuth components at high sintering temperatures, which results in poor sinterability and secondary phase formation [4,17]. Most studies have focused on the properties of unpoled KBT; however, to better understand and optimize KBT, measurements in the poled state are desirable. Additionally, there are no studies on the mechanical properties of this material.

In this work, the thermally induced phase transitions of both unpoled and poled KBT ceramics were investigated using X-ray diffraction, Raman and IR spectroscopy, differential scanning calorimetry (DSC), dielectric, and dynamic mechanical analysis techniques. To our knowledge, this is the first report on the temperature dependence of the structural, Raman, and mechanical properties of KBT in a poled state. The Raman and IR studies enabled the investigation of the local phenomena (lattice dynamics), due to their ability to probe small coherence length (spatial dimensions) and short timescales of physical phenomena, making them crucial for understanding macro-scale KBT properties. The combination of studies across various length scales provided a comprehensive understanding of this material and valuable insights into its complex behavior.

## 2. Experimental Methods

The KBT ceramics were prepared via a conventional solid-state synthesis route similar to that described in Suchanicz et al. [14]. Dried powders of high purity Bi_2_O_3_, (99.99%) TiO_2_ (99.9%) (all of Aldrich, Saint Louis, MO, USA) and KHCO_3_ were taken in stoichiometric ratios, and homogenized in a planetary ball mill for 24 h with ethanol. Unlike Suchanicz et al. [14], to allow careful adjustment of the stoichiometry during weighing, non-hygroscopic KHCO_3_ was used instead of hygroscopic K_2_CO_3_. In addition, to eliminate carbonate decomposition of Bi_2_O_3_, it was thermally treated at 400 °C for 9 h. The milled powders were then dried, uniaxially pressed into pellets at 100 MPa and calcined at 720 °C for 2 h and at 850 °C for 5 h with intermediate grinding. The products of the third calcination were again crushed into fine powders and pressed into pellets at 150 MPa. They were successively sintered at 910 °C for 1.5 h, at 1000 °C for 1.5 h, and at 1025 °C for 1.5 h. The products were then pulverized and milled again before further sintering. The powders were uniaxially pressed under a pressure of 250 MPa and finally sintered at 1030 °C for 5 h and at 1035 °C for 2 h. All calcination and sintering processes were carried out in an enclosed aluminum crucible, with pre-sintered KBT powder over the sample. The obtained ceramics were cream-colored and translucent, with a density greater than 97% of their theoretical value (measured using the Archimedes method) and a room-temperature resistivity of 10^13^ Ωcm.

The crystalline structure of KBT ceramics was examined by X-ray diffraction with a Philips X’Pert Pro MD diffractometer equipped with an MRI high temperature cell Standard Bragg-Brentano geometry was applied with K_α1,2_ radiation (the K_b_ line was suppressed by a Ni filter) from a Cu anode. Measurements were performed in air with a 0.017° step size in the 20–60° scanning range. Structural, qualitative, and quantitative phase analyses were made by Phillips X’Pert High Score Plus software (version 3.0.5), utilizing full-pattern fitting with the Rietveld method. This implementation of Rietveld was based on the source code of the program DBW3.2 from Wiles & Young (Georgia Institute of Technology, Georgia Tech.) [18].

X-ray photoelectron spectroscopy (XPS) was used to determine the precise chemical compositions and oxidation states of the elements on the sample surface. The XPS measurements were performed using Omicron Nano-Science equipment (Scienta Omicron, Uppsala, Sweden,), which features a hemispherical 128-channel Argus spectrometer (Scienta Omicron, Uppsala, Sweden,). Experiments were conducted at room temperature under ultra-high vacuum conditions, with pressure below 1.1 × 10^−6^ Pa. The spectra obtained were analyzed by the CASA XPS software (version 2.3.15) package, using Shirley background subtraction and the Gauss-Lorentz curve, GL (30), fitting using the least-squares method. To calibrate the results, a binding energy of 285.00 eV was established as the reference energy for the C1s signal (peak).

The dielectric measurements were performed using a GW 821 LCR Meter (Good Will Instrument, Taipei, Taiwan) in the temperature range from room temperature to 600 °C. An electric field of strength of 20 V cm^−1^ was applied. The data were collected at regular intervals with a of 0.1 °C step during heating and cooling, at a rate of 1.5 °C/min, using an automatic temperature controller Lake Shore 331 (Lake Shore Cryotronics, Westerville, Ohio, USA).

The differential scanning calorimetry (DSC) experiments were conducted using the DSC F3 Maia (Netzsch, Selb, Germany) apparatus. Measurements were done in the temperature range from −150 °C to 550 °C under an argon atmosphere at a flow rate of 30 mL/min. Measurements were performed with a rate of 10 °C/min.

The Raman spectra were obtained using a Bio-Rad FTS 6000 spectrometer (Bio-Rad Laboratories, Hercules, CA, USA) with a CCD detector (micro-Raman configuration, backscattering geometry). The 532 nm line of power 200 mW of a Nd:YAG laser system was chosen as the excitation beam. The spectra were collected with a resolution of 4 cm^−1^.

The IR reflection spectra at the range 200–900 cm^−1^ were recorded using a Bruker Vertex 70 v vacuum spectrometer (Vertex, King of Prussia, PA, USA). A Harrick Scientific External Reflection attachment (Seagull) (Harrick Scientific, Pleasantville, NY, USA) was employed. A total of 256 scans were recorded with a resolution of 4 cm^−1^.

Mechanical measurements were evaluated using a PerkinElmer model DMA8000 Dynamic Mechanical Analyzer (DMA) (PerkinElmer, Waltham, MA, USA), at a rate 1.5 °C/min, in three-point bending geometry at frequencies between 0.1 and 20 Hz.

The pyroelectric current of the previously polarized sample was measured by a quasi-static method on heating at a rate of 10 °C/min.

The poled state was achieved by applying electric fields of 5, 10, and 15 kV/cm to samples at 200 °C, followed by cooling down to room temperature while maintaining the applied field.

## 3. Results and Discussion

### 3.1. XPS Measurements

**Figure 1:**
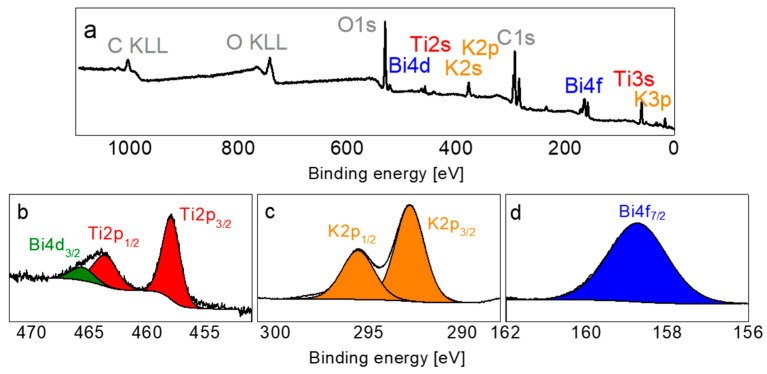
XPS overview spectrum (**a**) and high-resolution Bi4d and Ti2p (**b**), K2p (**c**), and Bi4f (**d**) core-level spectra of KBT ceramics.

Figure 1a presents the XPS survey spectrum recorded for the KBT samples. The samples exhibit high chemical purity, with only additional carbon detected, which originates from atmospheric adsorption. In Figure 1b–d, the high-resolution spectra for Ti2p, K2p, and Bi4f regions are presented. The Ti2p spectrum is somewhat complex due to the overlap of Ti2p and Bi4d signals [19]. It was deconvolved into three peaks, corresponding to Bi4d_3/2_, Ti2p_1/2_, and Ti2p_3/2_. The peak positions and the splitting energy, measuring 5.7 eV, are characteristic of the Ti^4+^ state [20,21]. The K2p spectrum (Figure 1c) shows a well-defined doublet, comprising K2p_3/2_ and K2p_1/2_ components, with a splitting energy of approximately 2.8 eV and an area ratio of 1:2. These features are typical of potassium in the K^+^ valence state [22,23]. In contrast, the characteristic doublet of Bi4f electrons (Figure 1d) indicates the Bi^3+^ state [24]. The average chemical composition calculated using the XPS spectra was estimated to be K_0.56_Bi_0.44_TiO_2.8_ and it is approximately close to the nominal composition. There is an excess of potassium and deficiency of bismuth and oxygen.

### 3.2. X-Ray Measurements

**Figure 2:**
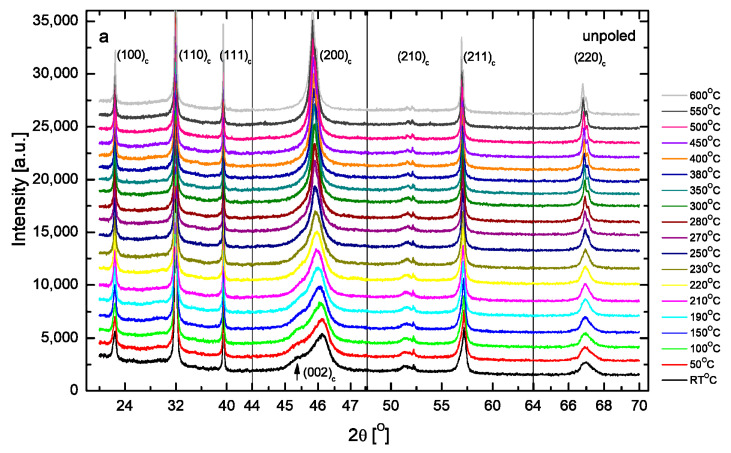

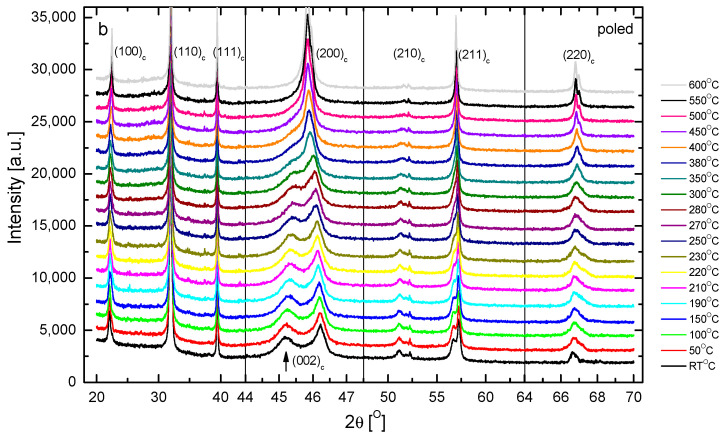
The temperature evolution X-ray diffraction patterns of (**a**) unpoled and (**b**) poled (15 kV/cm) KBT ceramics.

**Figure 3:**
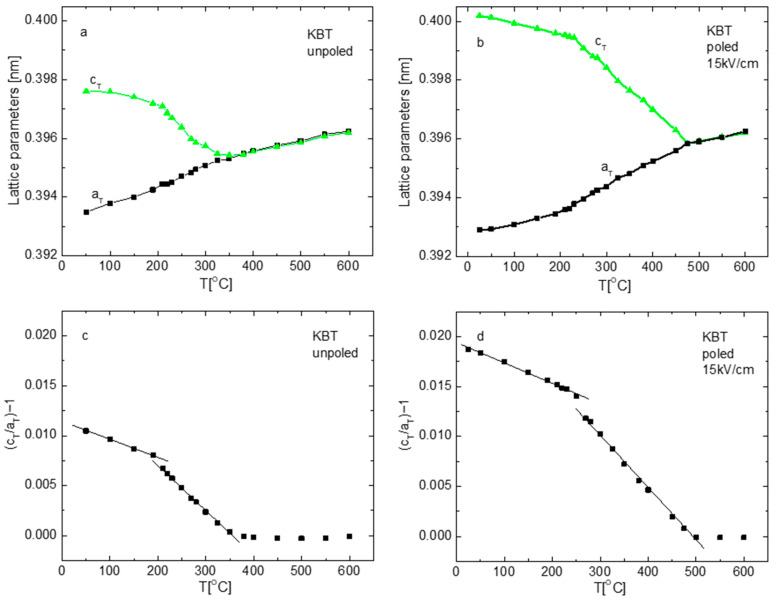
Lattice parameters (**a**,**b**) and tetragonality (**c**,**d**) of unpoled and poled KBT ceramics.

Figure 2 shows the temperature evolution of X-ray diffraction (XRD) spectra of KBT in unpoled and poled states. The cubic (200)_c_ reflection splits into (002)_c_/(200)_c_ reflections characteristic of the tetragonal phase. The peaks shift toward each other and gradually merge into the cubic single peak at ~400 °C for the unpoled state (see also Figure 3). The intensity of the (002)_c_ peak is smaller as compared to the (200)_c_ one (i.e., the intensity ratio of the (002)_c_/(200)_c_ peaks is small), which can be the result of a random distribution of unit cell orientation. It can be seen that a low-angle tail of the (200)_c_ peak still indicates the presence of some minority tetragonal phase (tetragonal polar regions) in the cubic matrix, even at 600 °C. Some broadening of the reflections of the cubic phase was observed, which also indicates the existence and temperature evolution of these regions. The temperature behavior of the majority of the reflections distinguished three characteristic regions. In the first range up to about 200 °C, a slow decrease in peak splitting and an increase in the maximum line intensities appears. Simultaneously, anomalies of lattice parameters and tetragonality are observed around this temperature (Figure 3).

In the second range (~200–400 °C), the intensity increases strongly in comparison to the first one. In the third one (~400–600 °C), the peaks become single and almost do not change intensity. These changes in the diffraction patterns indicate the presence of two phase transitions: a tetragonal-tetragonal (T-T) one at about 200 °C and a tetragonal-cubic (T-C) one at about 400 °C. Computer analysis confirmed these qualitative predictions. From the lowest temperatures, the tetragonal phase (P4mm) exists up to approximately 400 °C, while above this temperature, the cubic phase (Pm$\bar{3}$m) exists. At 200 °C, no change in the point-group symmetry is detected. We only observe a gradual evolution of the lattice parameters, with the a and c periods slowly converging over the temperature range from RT to 200 °C (Figure 3a). For the poled state (15 kV/cm), the tetragonal (002)_c_/(200)_c_ peak splitting is more pronounced (the intensity ratio of the (002)_c_/(200)_c_ peaks has an equal 1:2 characteristic for the tetragonal phase) in comparison to the unpoled one, and the splitting disappears at a temperature of approximately 450 °C instead of 400 °C for the unpoled one. An increase in intensity of the (002)_c_ peak compared to (200)_c_ is expected because poling causes a preferred orientation of the unit cell within the field direction and reorientation of the ferroelectric domains. The tetragonal-tetragonal phase transition is also shifted from about 200 °C to about 220 °C. As the 180° domain reversal does not affect the XRD intensities, the change in the intensity ratio of the (002)_c_/(200)_c_ peaks only reflects the 90° domain reorientation. The calculated percentage of 90° domain reorientation by electric field (15 kV/cm) is about 21%. E-poling also causes a change in lattice parameters, which leads to a drastic increase in tetragonality and distinguishes the temperature of 270 °C as a characteristic one, about which a clear anomaly of these parameters is visible (Figure 3). In general, these results indicate the remarkable rearrangement of the crystal structure and show that the electric field transforms the cubic phase into a tetragonal (ferroelectric) one, and extends the temperature range over which the latter phase exists.

**Figure 4:**
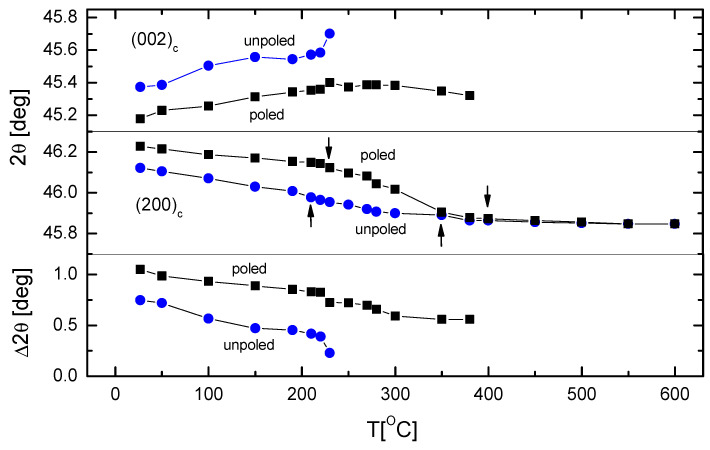
The temperature variation in the 2Θ positions and position difference Δ2Θ of (002)_c_ and (200)_c_ reflections of unpoled and poled (15 kV/cm) KBT ceramics. The arrows show a slope change in the 2Θ vs. temperature curves.

For closer inspection, the 2Θ-position and the 2Θ-position difference of the (002)_c_ and (200)_c_ peaks are plotted against temperature in Figure 4. Apart from lower angle shift, the 2Θ-T curve for the (200)_c_ peak also undergoes a slope change at ~200° and at ~380–400 °C for the unpoled state (three different straight lines can be fitted to the points). The lower-angle shift of the Bragg peaks indicates a gradual increase in unit cell volume with increasing temperature; however, the slope changes of 2Θ-T curves indicate structural transformations. The symmetry changes which occurred in KBT are from the room-temperature tetragonal phase to a high-temperature cubic phase, and additionally, between tetragonal phases at about 200 °C. Considering the phase transitions sequence, the slope changes of 2Θ-T curves can be attributed to the tetragonal-tetragonal and tetragonal-cubic phase transitions. For the poled sample, the slope changes of 2Θ vs. temperature curves are shifted towards higher temperature, which further show that the electric field transforms the cubic phase into a tetragonal (ferroelectric) one and extends the temperature range in which the tetragonal phase exists. In addition, in both unpoled and poled samples, a shift in the position of the (002)_c_ reflection to larger lattice spacings is observed, while a smaller shift of the peak position of the (200)_c_ reflection to lower lattice spacings is also seen. The position of the (002)_c_ peak for the unpoled sample shifts toward larger lattice spacing with increasing temperature, with an anomaly at about 200 °C. For the poled sample, it also shifts in the same manner, with an anomaly at about 220 °C. As the temperature increases, the 2Θ-position difference of the (002)_c_ and (200)_c_ peaks decreases, with an anomaly at about 200 °C for the unpoled state and at about 220 °C for the poled state.

### 3.3. Dielectric Properties

**Figure 5:**
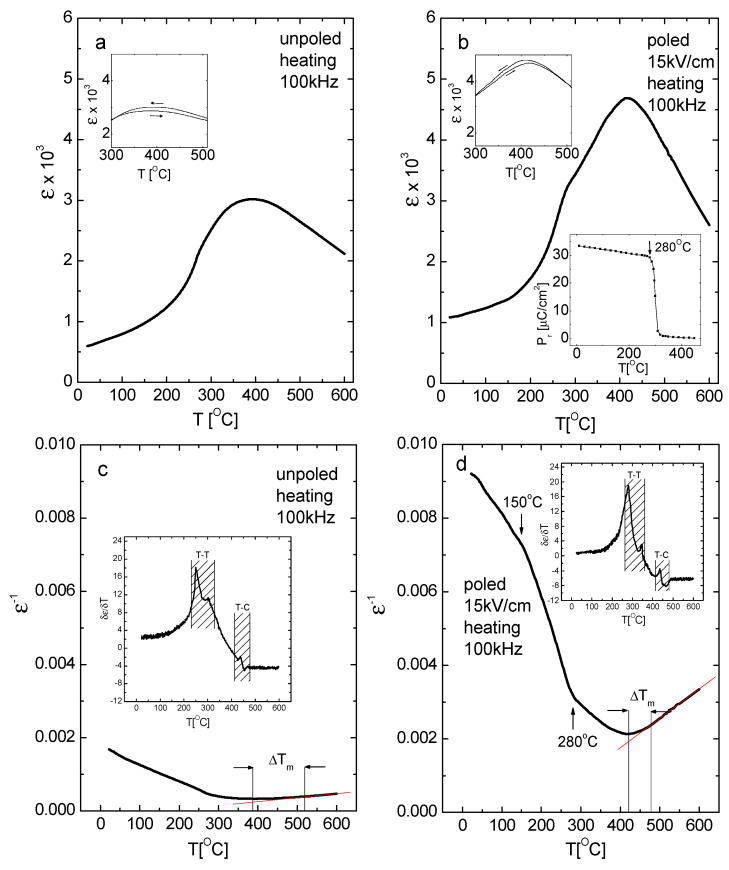
Temperature evolution of electric permittivity ε (**a**,**b**), ε^−1^ (**c**,**d**), and δε/δT vs. T (inserts of (**c**,**d**)) of unpoled and poled (15 kV/cm) KBT ceramics on heating (f = 100 kHz). Upper inserts in Figure 3a,b present the temperature dependence of ε on heating/cooling, showing temperature hysteresis. The lower insert of (**b**) shows the temperature evolution of remnant polarization of KBT ceramics.

The temperature evolution of the electric permittivity ε(T) of unpoled and poled (15 kV/cm) KBT measured during heating is shown in Figure 5a,b. The ε(T) curves for the unpoled and poled states differ. In the unpoled state, after a rather slow increase in ε up to about 215 °C, there is a faster increase up to about 270 °C, and then it enters a “diffuse region”, where it increases slowly up to T_m_ ≈ 390 °C, where the maximum occurs. Above T_m_, ε decreases gradually.

Conversely, in the poled state, after a slow increase in ε up to about 150 °C, there is a faster increase up to about 280 °C (see also Figure 5d), and then there is slower growth up to T_m_ ≈ 410 °C, where the maximum is observed. Above T_m_, ε decreases more rapidly than in the unpoled state. Overall, dielectric anomalies are sharper and shifted to higher temperatures after poling compared to unpoled samples. In both cases, the ε(T) evolution reveals characteristic temperatures visible in XRD measurements.

Both unpoled and poled states exhibit thermal hysteresis of the electric permittivity (shown in inserts in Figure 5a,b), indicating a first-order phase transition. It is expected that applying an electric field at a high temperature (200 °C), followed by cooling to room temperature in the presence of the field, modifies both the crystal structure—transforming the cubic phase into a tetragonal one—and the domain structure, increasing the proportion of monodomain-like state. These changes affect the inter-phase boundaries and density of domain walls (twin walls). The structural and domain states of KBT can be specifically manipulated by controlled poling, i.e., by applying a poling field of varying strength at different temperatures or with varying cooling durations. In particular, the temperature notably influences the poling process due to a decrease in coercive field and reduced lattice distortion at elevated temperatures, which facilitates easier modification of the crystal structure and increases the fraction of domain reorientations. Modifying the crystal structure and domain state via an electric field provides a practical means to tailor the material’s physical properties.

At high-temperatures, ε(T) follows the Curie-Weiss law, as shown in Figure 5c,d. Below about 520 °C and 470 °C, ε(T) begins to deviate from the Curie-Weiss law for the unpoled and poled states, respectively. The E-poling extends the temperature range in which ε(T) adheres to the Curie-Weiss law (i.e., ΔT_m_ = T_B_ − T_m_ decreases, where T_B_ is the Burns temperature, below which ε^−1^ does not follow a linear trend).

To better understand the nature of the tetragonal-tetragonal and tetragonal-cubic phase transition, the δε/δT versus T plots are presented in inserts of Figure 5c,d. The abrupt inflection points near both the tetragonal-tetragonal and tetragonal-cubic (T_m_) temperatures seem to be more sharp for the poled state compared to the unpoled state. Since such inflections mark first-order behavior, this suggests that the transition tends to become more first order after E-poling. This implies that electric poling enhances the order within KBT (i.e., the electric ordering is improved by the electric field).

It is noteworthy that the ε(T) features of poled samples remain stable and essentially unchanged after multiple heating/cooling cycles up to T_d_ = 270–280 °C, where a distinct anomaly exists (Figure 5b,d). This stability is further supported by the behavior of the remnant polarization P_r_ (shown in lower insert in Figure 5b). P_r_ decreases slightly up to about 275 °C (the onset of depoling behavior), followed by a more abrupt change until roughly 305 °C, and then decreases very slowly up to approximately 450 °C. Therefore, the depolarization temperature (T_d_) of KBT exceeds that of the lead-free NBT analogue by over 100 °C, which is significant for practical applications. The temperature-dependent evolution of remnant polarization indicates that polar behavior persists across a wide temperature interval, up to about 450 °C.

### 3.4. Differential Scanning Calorimetry

**Figure 6:**
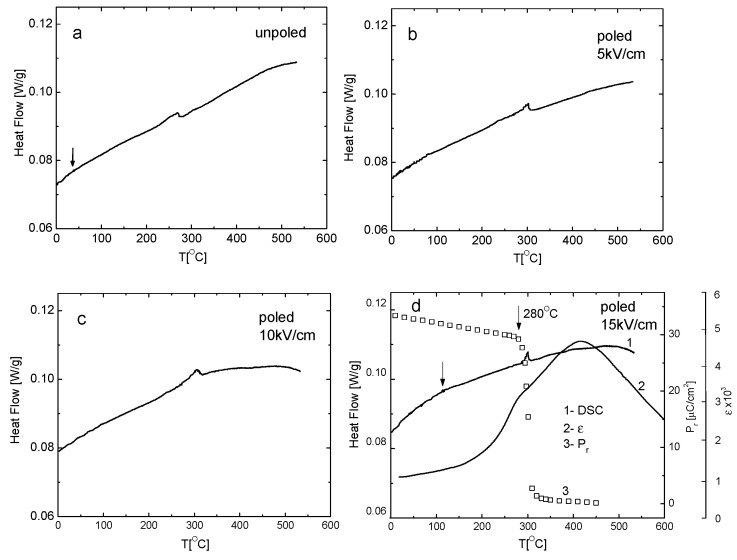
Heat flow of (**a**) unpoled, (**b**) poled 5 kV, (**c**) poled 10 kV and (**d**) poled 15 kV for KBT ceramics on heating. The arrow shows a broad anomaly in the low-temperature range. (**d**) The DSC(T) curve of the poled (15 kV/cm) sample (1) also presents temperature variation of electric permittivity ε (2) and remnant polarization P_r_ (3) for comparison.

Figure 6 illustrates the temperature dependence of heat flow (DSC) during the heating process for both unpoled and poled samples. A distinct endothermic peak is observed for both states. This peak for unpoled state occurs at approximately 275 °C. After poling at an electric field intensity of 15 kV/cm, the DSC anomaly shifts to a higher temperature, reaching approximately 295 °C. Consequently, this heat effect takes place within the temperature range of the depoling phenomena, where a distinct decrease in tetragonality (Figure 3b) and an anomaly of electric permittivity exists (Figure 6d). Additionally, the DSC peak is sharper and larger (with an increased area under the peak) for the poled sample compared to the unpoled one. Since this area reflects a free-energy difference between the two phases, it indicates that the electric field enhances the stability of both phases. In addition to the main anomaly, a broad peak of the DSC curves (indicated by an arrow) appears, which seems to shift towards higher temperatures as a result of electric field poling.

### 3.5. Raman Spectroscopy

**Figure 7:**
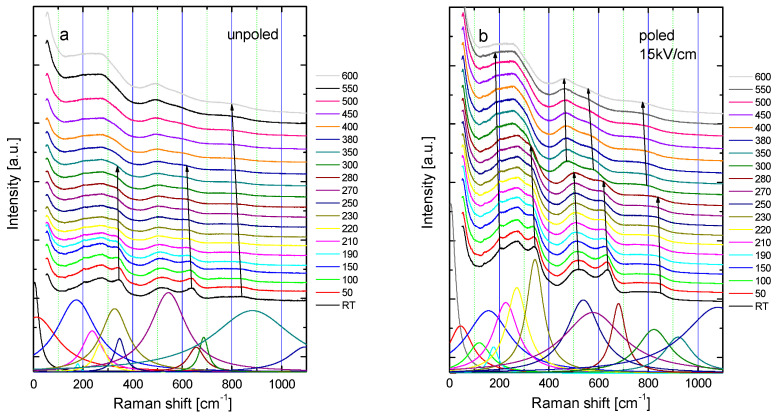
The temperature evolution and deconvolution Raman spectra of (**a**) unpoled and (**b**) poled KBT ceramics. Solid arrows guide the variation in the phonons line center with temperature showing a distinct change in frequency of the 520, 615, and 836 cm^−1^ modes in the temperature interval of depoling phenomena (**b**).

Raman spectra of KBT ceramics at various temperatures are presented in Figure 7. Multi-Lorentzian oscillator functions were utilized to fit Raman spectra, as shown in Figure 7.

The broad modes in wavenumber observed around 150–300 cm^−1^ indicate an increase in short-range polarization, in contrast to sharp features typical of long-range polar order. The spectra can be divided into three main regions: (1) the bands at about 100–350 cm^−1^ originating from K-O and Ti-O vibrations, (2) the bands near 500 and 650 cm^−1^ dominated by Ti-O octahedral vibrations and rotations, and (3) the bands above 700 cm^−1^ related to oxygen vibration and the presence of oxygen vacancies [25]. Modes associated with A-site cations (K^+^ and Bi^3+^) are likely to occur predominantly below about 200 cm^−1^. As temperature increases, the two splitting modes in the 280–370 cm^−1^ range first broaden, and eventually (around 400 °C) one mode at about 280 cm^−1^ persists. This suggests a change in symmetry towards a structure with fewer Raman active modes, linked to the gradual disappearance of both the tetragonal phase and the long-range ferroelectric state (the mode near 280 cm^−1^ is also related to the ferroelectric phase transformation). The mode at approximately 280 cm^−1^ develops throughout the investigated temperature range and remains quite complex even at 600 °C. Similar behavior is observed in the modes at about 520 and 836 cm^−1^, implying that the symmetry is not purely cubic at temperatures well above T_m_. These modifications reflect the shift in average crystal symmetry seen in the XRD data and show that polar-active Ti-O vibrations persist within this temperature range. At the same time, oxygen vibrations and rotations are also present in the high-temperature cubic phase, which exhibits a short correlation length. It is vital to note that, according to X-ray results, above approximately 400 °C, the large-length scale average structure of KBT is cubic (Pm$\bar{3}$m). When the temperature exceeds T_m_, the cubic phase gradually develops, causing the Raman peaks to broaden, with modes overlapping and becoming indistinct during the fitting process. As a result, the fitting outcomes may be affected by other phonon modes and cannot be clearly separated. The disorder of A-site cations in KBT necessitates the existence of the chemical 1:1 order in the K/Bi sublattice (chemically ordered nanoregions) [14]. Another reason for the observed first-order Raman spectra in the cubic phase is the presence of unstable polar regions (most likely tetragonal), whose existence may fluctuate over their lifetime within this temperature range [14]. Since Raman spectroscopy is sensitive to the local symmetry, it can detect these regions. Furthermore, the presence of polar regions in the paraelectric phase of perovskites has also been reported and interpreted as pre-transitional effects (precursor dynamics), resulting from interaction between polar and elastic areas [26,27,28].

It is expected that applying an electric field induces changes in the distances and displacements of ions. These modifications alter force constants, thereby affecting vibration conditions. In the poled state (Figure 7b), most modes appear sharper and more symmetric, their intensities increase, and they shift towards lower wavenumbers, indicating a change in crystal structure and/or ferroelectric order. This Raman behavior suggests the development of long-range polar order and an increase in the degree of ordering after E-poling.

**Figure 8:**
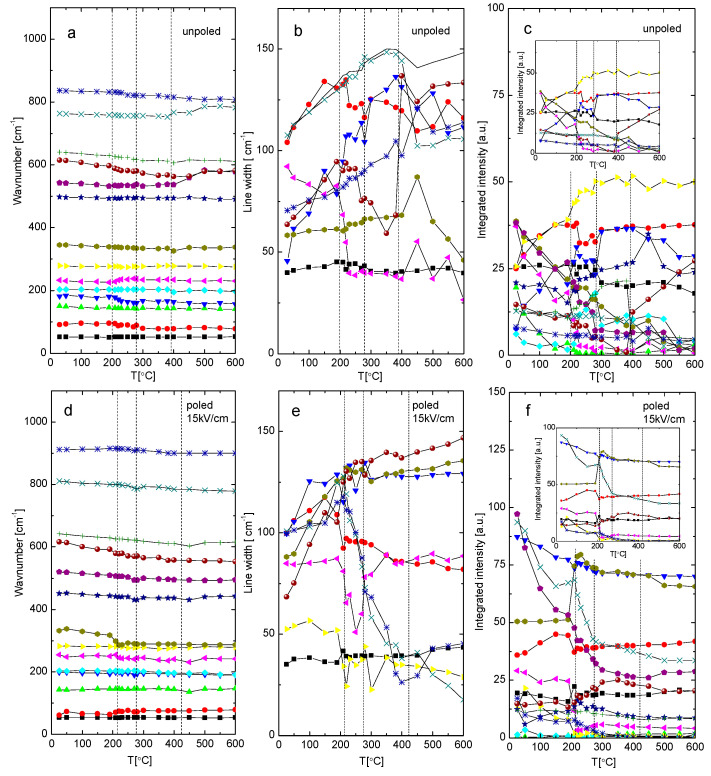
The temperature variation of wavenumber, line width, and integrated intensity of (**a**–**c**) unpoled and (**c**–**e**) poled (15 kV/cm) KBT ceramics. For clarity, (**b**,**e**), and inserts in (**c**,**f**) show selected modes. The dashed lines correspond to the T-T phase transition point, temperature of depoling, and T-C phase transition point detected in X-ray, dielectric, and pyroelectric measurements.

The distinct shift in the frequency of the 520, 615, and 836 cm^−1^ modes occurs in the poled state at about 280–290 °C (Figure 7b and Figure 8d), aligning with the temperature range where depoling phenomena, as previously discussed, are observed. This includes a reduction in tetragonality (Figure 3b), an anomaly in electric permittivity (Figure 5b,d) and DSC (Figure 6d), and a rapid change in remnant polarization (insert in Figure 5b and Figure 6d). These facts clearly demonstrate that local-scale processes significantly influence macroscopic properties.

The disorder of KBT arises from the presence of two different ions, K^+^ and Bi^3+^ at the A-site. Beyond differences in charge, these ions also differ in several respects: (1) the ionic radius is 1.64 Å for K^+^ and 1.17 Å for Bi^3+^, with coordinator number XII and VIII, respectively; (2) the K-O bond is nearly ionic, but the Bi-O bond is predominantly covalent; (3) the bond lengths vary; and (4) the electronegativity is 0.82 for K^+^ and 2.02 for Bi^3+^. These differences create notably distinct local environments, causing distinctly different local environments for K and Bi. Due to these substantial differences, the partial local ordering of A-site cations—forming chemically ordered nanoregions—is plausible [14] in accordance with theoretical predictions [29]. Both Bi and K easily evaporate, leading to local deficiencies or excesses in the cation sublattice. The difference in ion sizes and valences causes local lattice stresses, promotes fluctuations in occupancy at lattice sites, and leads to dynamic (disordered) shifts and local inhomogeneities. Bi ions have quite a complex electronic structure and can appear as Bi^3+^ and Bi^5+^. Therefore, we can speculate that they can be incorporated into different places within the structure. If that is the case, it would mean that Bi can modify the density of defects, especially by reducing the density of oxygen vacancies, thereby supplying the structure with electrons. Therefore, one cannot exclude Bi disorder in the KBT structure; however, it is not acting negatively, but rather positively, on the polar properties presented in our paper.

For closer examination of the local structure state, the temperature evolution of the peak position, full width of half maximum (FWHM), and intensity of the Raman modes derived from spectral deconvolution are presented in Figure 8. Overall, the Raman parameters exhibit an almost classical temperature variation: (1) the wavenumber decreases and modes broaden, (2) FWHM increases (while remaining relatively small, indicating a large coherence length), and (3) for most modes integrated intensity decreases, suggesting the first-order character of these modes. The modes change non-monotically, with sudden shifts around phase transitions, particularly around the tetragonal-tetragonal phase transition; smaller changes occur during the tetragonal-cubic phase transition. This includes a significant change in peak intensities and abrupt jumps in their positions and widths, likely related to the different ionic arrangements associated with changes in crystal structure. The variations in the Raman line parameters are more noticeable in the poled sample, due to decreased structural disorder caused by the electric field. At least three Raman modes, 91, 182, and 615 cm^−1^, show softening-like behavior on approaching T-T phase transition (Figure 8a).

Above this temperature, frequencies of these modes remain nearly constant, indicating the displacive character of this transition. In the poled state, modes 91, 233, 345, and 615 cm^−1^ demonstrate similar softening-like behavior near this transition (Figure 8d). The modes 641 (possibly also 615), 345, and 204 cm^−1^ display softening-like behavior approaching the C-T phase transition (Figure 8a), with modes 641 (possibly also 615), 345, 233, and 182 cm^−1^ showing similar tendencies in the poled state. Generally, applying an electric field causes the wavenumber to shift towards lower frequencies, decreases the FWHM, and increases the integrated intensity. Notably, in the poled state, an anomaly in the temperature evolution of some modes (including the 280 cm^−1^ mode related to Ti-O vibrations) appears near the depolarization temperature T_d_. Based on the anomalies in the temperature evolution of the wavenumber, linewidth, and intensity, phase transition temperatures were estimated. For the unpoled sample, these temperatures are ~195 °C and ~375 °C for the tetragonal-tetragonal and tetragonal-cubic phase transitions, respectively (Table 1). For the poled sample (15 kV/cm), the estimates are higher, around 210 °C and 410 °C.

**Figure 9:**
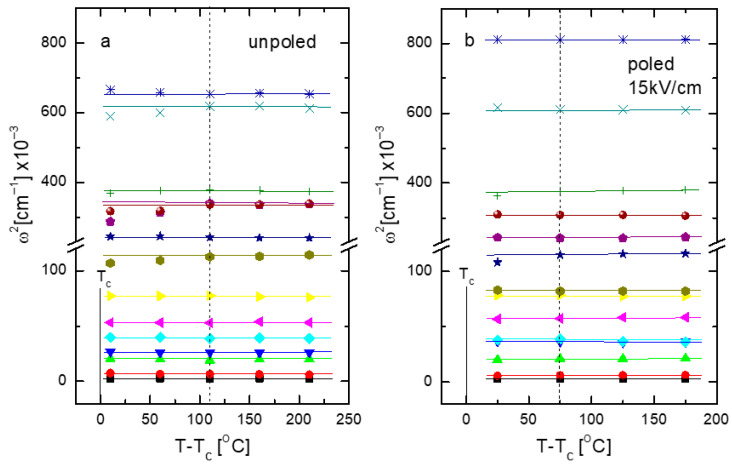
Temperature dependence of the modes’ squared frequency for (**a**) unpoled and (**b**) poled KBT ceramics. The dashed line designates the temperature of the onset of precursor dynamics.

As mentioned earlier, some Raman modes show changes resembling softening at characteristic temperatures (Figure 7 and Figure 8a,d) derived from X-ray and dielectric measurements, as previously described. This suggests that the phase transition is mainly of displacive character. According to Cochran’s law, the square of the soft-phonon frequency should be a linear function of temperature: ω^2^~(T-T_c_). Figure 9 shows the temperature variation of the squared frequency of the modes with a linear fit (the same symbols are used for particular modes, as for Figure 8). In general, this behavior is observed for low-frequency modes; however, for high-frequency modes, a divergence from the linear ω^2^(T) function appears at about 500 °C and 450 °C for the unpoled and poled (15 kV/cm) states, respectively (Figure 9). These temperatures roughly correspond with Burn’s temperatures obtained from the Curie-Weiss law and also agree with theoretical predictions made by Bussmann-Holder A. et al. [26,27]. This behavior can be connected in a first approximation with a non-homogeneous distribution of chemically ordered nanoregions and/or with local strain associated with them. Referring once again to the theoretical considerations [26,27,28], precursor dynamics in the paraelectric phase are not related to inhomogeneity but to acoustic-optic mode coupling as an inherent effect. From this perspective, the derived temperatures arise from the presence of polar regions in the high-temperature range and the shift of these regions from stable to dynamic (not with their disappearance) as temperature increases. As temperature decreases, stable polar regions start interacting with each other and grow in size, eventually leading to a phase transition at T_c_.

It is known that when the tolerance factor is approximately one, a stable cubic phase can form. When the tolerance factor is significantly greater or smaller than one, it indicates the possible presence of octahedral tilts and their deformation. This deformation may lead to instability in phonons within the Brillouin zone. Because the calculated tolerance factor for the investigated material is 1.02, i.e., very close to one, one cannot expect distinct instability of phonons at the Brillouin zone. In principle, the Raman scattering does not “see” the modes from the Brillouin zone. However, this can happen due to the presence of defects and disorder. Then, the crystal lattice disturbances can “loosen” the selection rules and enable the observation of phonons outside the Γ point, usually at high values of the wave vectors. The result is that the Raman spectrum is much broader than the “normal Raman line”. Therefore, we cannot exclude that our Raman spectra at high wavenumbers are influenced by the modes from the Brillouin-zone instability.

### 3.6. IR Spectroscopy

**Figure 10:**
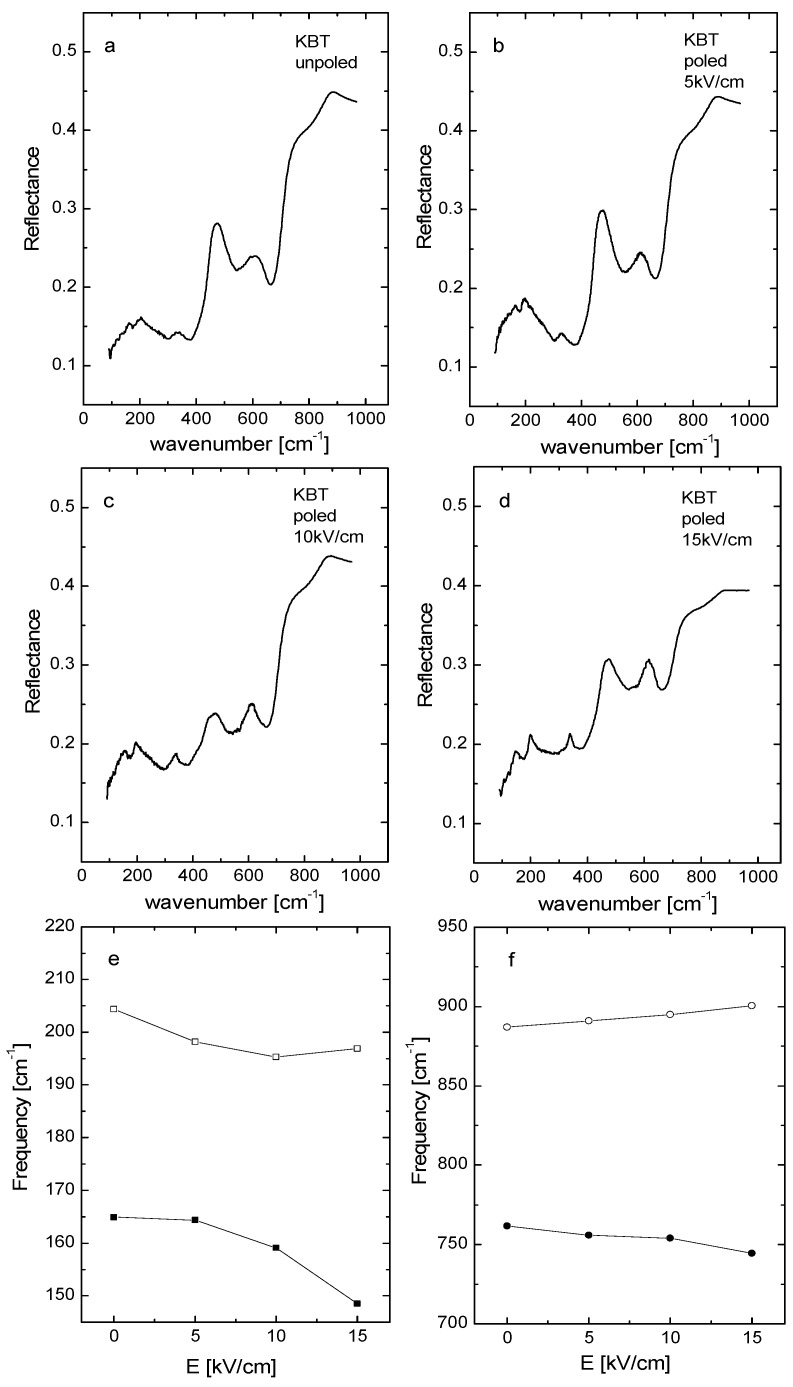
IR spectra of unpoled (**a**) and poled (**b**–**d**) and frequency variation trends of some typical IR-active modes with increasing electric field strength (**e**,**f**) of KBT ceramics.

Figure 10a–d show the IR spectra of KBT for unpoled and poled states. The three reflection bands, located at approximately 100–200, 200–600, and 650–900 cm^−1^, are visible. The lowest band, linked with the Bi-TiO_6_ vibrations, lies outside of our experimental conditions. The modes near 100–200 cm^−1^ are related to vibrations of the A-site ion (K) in TiO_6_. The band around 200–600 cm^−1^ corresponds to Ti-O vibrations. The bands at 650–800 cm^−1^ primarily result from the octahedral stretching vibrations. Generally, the reflectivity increases gradually in the low wavenumbers, and its bands become more symmetric due to E-poling, which enables dynamic order. Additionally, the electric field causes a shift of the low-wavenumber bands towards lower wavenumbers, with some redistribution of their intensity observable (Figure 10b–f). These changes indicate a significant rearrangement of the lattice structure, which aligns with the Raman results. The increase in unit cell volume resulting from E-poling weakens interactions between A-site ions, TiO_6_ octahedra, and Ti and O ions, leading to a shift of the corresponding IR bands towards lower wavenumbers.

The Raman- and IR-active modes, along with their evolution under the electric field, are summarized in Table 1. This list highlights the origin of phonon modes and clearly reflects the influence of E-poling on lattice vibrations.

### 3.7. Mechanical Properties

**Figure 11:**
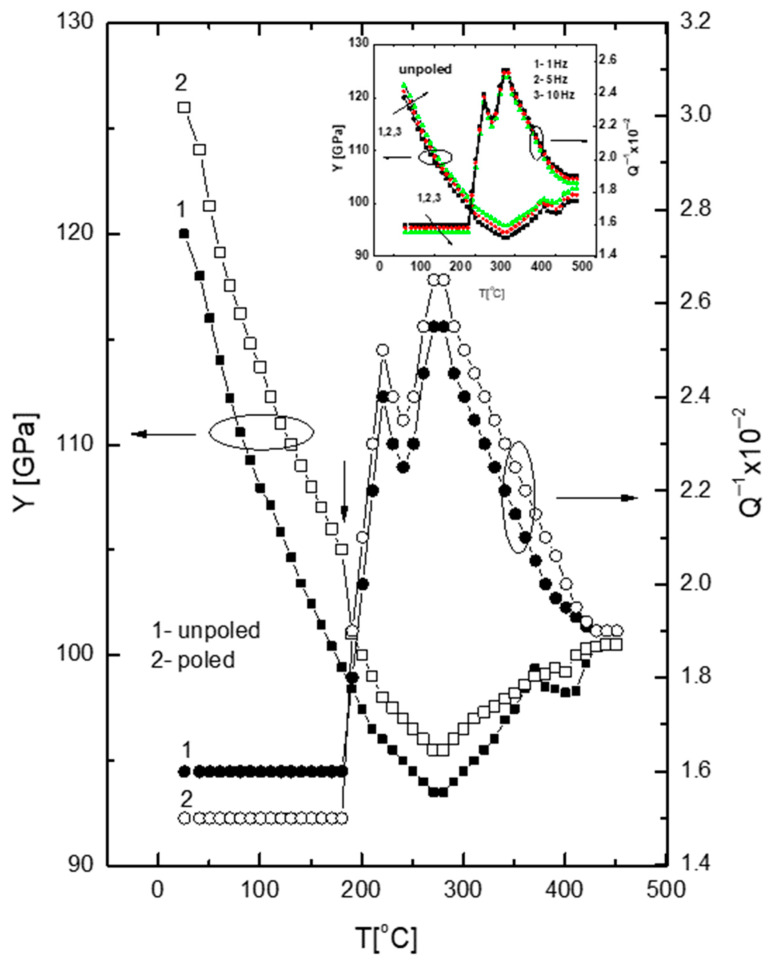
The temperature evolution of Young’s modulus Y and internal friction Q^−1^ of unpoled and poled (15 kV/cm) KBT ceramics on heating. The arrow shows a local anomaly of the Young’s modulus of the poled sample. Insert shows temperature/frequency dependence of Y and Q^−1^ on heating, showing a small frequency dispersion in a wide temperature range including the phase transitions.

Figure 11 depicts the temperature dependence of Young’s modulus (Y) and the internal friction (Q^−1^) for unpoled and poled samples. The Y(T) curve exhibits two anomalies: (1) at approximately 270 °C (minimum) and (2) the small peak at about 370 °C. The first anomaly corresponds to depolarization phenomena described earlier, and indicates increased domain wall movement before the tetragonal-cubic phase transition.

The second anomaly aligns with the maximum electric permittivity. A detailed examination of Figure 11 reveals a minor anomaly at approximately 200 °C (marked by an arrow), which is associated with the tetragonal-tetragonal phase transformation. The Y drops from ~120 to 93 GPa in the temperature range RT-270 °C, indicating elastic softening similar to that observed in improper ferroelastic transitions. This softening primarily results from the coupling between spontaneous strain and the order parameter, which is linked to a specific tilt angle of the oxygen octahedron. At a constant spontaneous strain level, the restoring force resisting octahedral tilting weakens to the point that the material softens [30]. Additionally, the movement of twin walls contributes to this elastic softening. An anomaly resembling an elastic softening-like anomaly can also be observed near 400 °C, which corresponds to the tetragonal-cubic phase transition.

The Q^−1^(T) curve presents three anomalies (1) around 215 °C, coinciding with anomalies in ε and Y and related to the tetragonal-tetragonal phase transition, (2) around 270 °C, matching the epsilon anomaly and Y minimum, and corresponds to depoling effect, associated with increased of domain walls movement before the tetragonal-cubic phase transition, and (3) near 370 °C, corresponding to the maximum of both ε and Y, linked to the tetragonal-cubic phase transition. It is plausible that electromechanical interaction between the tetragonal matrix and chemically ordered matrix, as well as between the cubic matrix and tetragonal regions, significantly influence the evolution of mechanical parameters Y and Q at low and high temperatures, respectively. It is noteworthy that twin walls between 90° domains in the tetragonal symmetry of KBT are both ferroelectric and ferroelastic in nature, as multiaxial ferroelectrics display simultaneous weak ferroelasticity [31,32]). The former are sensitive to the electric field, while the latter respond to stress. The elastic strain field is weaker and longer-ranging than the electric field, which, although stronger, is relatively short-range. This loss mechanism in KBT is likely to involve mobile interfaces between ferroelastic domains. The anomalies and frequency dependence of Q^−1^ are primarily governed by the ferroelastic aspects of the tetragonal-tetragonal and tetragonal-cubic phase changes, possibly resulting from the relaxation of twin walls between 90° domains and mobile interfaces between coexisting phases.

Excellent agreement between the transition temperatures obtained using XRD, DSC, dielectric, Raman, and mechanical measurements is clearly evident, as shown in Table 2. Note that the temperature of the tetragonal-cubic phase transition obtained from Raman studies is lower in comparison to that obtained using other measurements. This difference can be mainly due to the varying scales of changes detected by the techniques and the stress effect, which can shift the phonon frequencies [33,34]. X-ray, DSC, mechanical, and dielectric responses relate to macroscopic structural changes, whereas the Raman technique provides information about microscopic (local scale) structural changes. Apart from the different local stress fields within individual grains, there are at least two reasons for the existence of stress in KBT: (1) the difference in the ionic radius of ions occupying the A-site and (2) the existence of chemically ordered nanoregions and polar regions across a wide temperature range. These stresses can lower the temperature at which the tetragonal-cubic phase transition occurs in Raman studies. Some differences in the phase transition temperatures compared to previous studies may be due to variations in technological conditions used by different authors.

**Figure 12:**
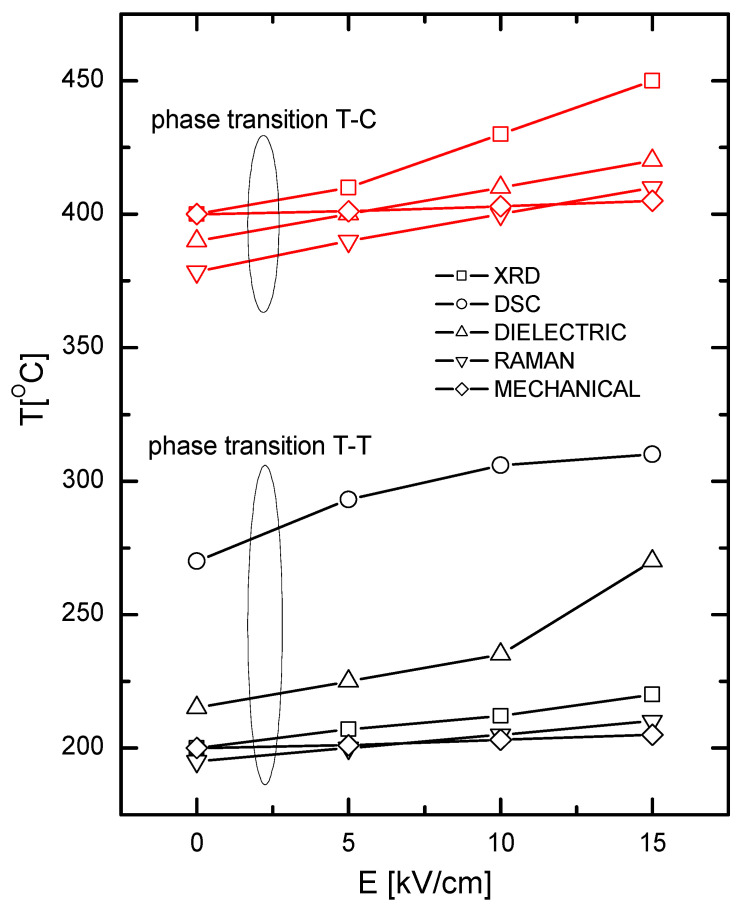
Electric field dependence of transition temperatures for tetragonal-tetragonal and tetragonal-cubic phases of KBT ceramics.

The transition temperatures from our measurements are plotted against the electric field intensity in Figure 12. As shown, increasing the electric field intensity has a consistent effect on the temperature for both the tetragonal-tetragonal and tetragonal-cubic phase transitions, displaying an almost linear increase. Excellent agreement between the transition temperatures obtained via different measurement techniques is again clearly evident.

To enhance properties of the perovskites, dopant strategies are commonly employed. We have demonstrated that E-poling is an effective method for achieving a similarly stable enhancement effect.

## 4. Conclusions

Structural, dielectric, calorimetric, and mechanical measurements have been conducted to study the phase transformations of unpoled and poled KBT. Two structural phase transitions are identified: tetragonal-tetragonal around approximately 200 °C and tetragonal-cubic at about 400 °C. The tetragonal-cubic transition is smooth in the unpoled state. X-ray investigations reveal the transformation of the cubic phase into the tetragonal phase induced by an electric field. The first-order Raman scattering is observed throughout the entire measured temperature range, even in the paraelectric cubic phase. We propose that Raman spectra in the cubic phase originate from both the 1:1 K/Bi chemically ordered nanoregions and from polar regions of tetragonal symmetry. It was found that tetragonal-tetragonal and tetragonal-cubic phase transformations are accompanied by significant elastic softening, which correlates with anomalies of polarization, DSC, electric permittivity, and Raman spectra. The results clearly indicate coupling between ferroelectric and elastic (strain) properties and that phenomena occurring on a local scale have an impact on macroscopic properties.

It was demonstrated that E-poling is an economical, rapid, and effective method for modulating the material’s properties. Furthermore, the effect of E-poling remains stable over a wide temperature range, up to T_d_ = 280 °C, making it relevant for practical applications. This study offers a clear understanding of the electric field structure-function relationship within the KBT heterophase coexistence system, potentially supporting the development of high-performance piezoelectric materials.

This research did not receive any specific grant from funding agencies in the public, commercial, or not-for-profit sectors.

## Figures and Tables

**Table 1:** The comparison of Raman-active and IR-active vibration modes for unpoled and poled KBT ceramics.

A Position Cation Translation Mode (cm^−1^)	A-B-O Bending Vibration (cm^−1^)	O-B-O Stretching Vibration (cm^−1^)	The Irreducible Coupling Mode (cm^−1^)
Raman			
0 kV/cm 51 91 150 182	204 233 278 345	497 541 615 641 764	836
5 kV/cm 50 85 148 196	203 220 274 347	498 540 614 642 805	858
10 kV/cm 49 86 146 183	203 235 274 346	497 542 620 643 807	891
15 kV/cm 48 62 143 198	202 254 282 332	450 520 616 642 811	920
IR			
0 kV/cm 165	204 233 335	473 608 763	885
5 kV/cm 164	199 230 332	472 610 759	886
10 kV/cm 160	197 226 329	471 613 761	889
15 kV/cm 149	199 224 327	472 615 751	892

**Table 2:** Temperature of tetragonal-tetragonal (T-T) and tetragonal-cubic (T-C) phase transitions of unpoled KBT ceramics obtained by different techniques.

Technique	Phase Transition	
	T-T [°C]	T-C [°C]
XRD	200	400
DSC	275	-
Dielectric	-	390
Raman	195	375
Mechanical	200	400

## Data Availability

The original contributions presented in this study are included in the article. Further inquiries can be directed to the corresponding authors.
